# Prospective Drivers of Nurses’ Partial or Complete Retirement Seven Years Later: Work Ability and Physical Functioning Going against the Tide of Age

**DOI:** 10.3390/ijerph191811159

**Published:** 2022-09-06

**Authors:** John Rodwell

**Affiliations:** Department of Management & Marketing, Swinburne University of Technology, Melbourne, VIC 3122, Australia; jrodwell@swin.edu.au

**Keywords:** age, work ability, bridge employment, physical functioning, nurses, retirement

## Abstract

In the context of a nursing shortage in many developed countries, one option for retaining the expertise of older nurses in the workforce is to better manage the retirement of nurses. This study will investigate life course predictors of actual retirement behavior, whether partial or complete, for older working nurses over a seven-year span. Data were analyzed from a quantitative, longitudinal survey of 178 Australian nurses aged 45 years and older working at Time 1 (2012), who responded seven years later (Time 2). Age predicted both forms of retirement behavior, confirming the strength of social normative pressures toward retirement. Moving against this social normative tide toward retirement were the predictors of partial retirement such as job satisfaction, physical functioning and work ability. In turn, working part-time was the only predictor, beyond age, of complete retirement. Nurse and health service managers could support nurses to stand against the tide of social normative pressures (until such social norms change) by managing the nurses’ physical functioning and work ability, including by more explicitly managing the impacts of chronic diseases in the workplace, not least so as to retain their nurses in the context of nurse shortages.

## 1. Introduction

Many developed countries, including Australia, are facing nursing shortages as part of a global shortfall of nurses [1]. One avenue for addressing these shortages is to retain the expertise of older nurses in the workforce by either delaying complete retirement [2] or by increasing more flexible work options such as by enabling partial retirement, also known as bridge employment, which is a form of employment that “bridges” from pre-retirement employment to full retirement [3].

The possible staffing benefits gained from a better understanding of the drivers of nurse retirement are substantial. For example, in Germany and the United Kingdom only a small proportion of nurses continue in the workforce until age 60 [4]. In Australia a staffing “cliff” may be emerging because the proportion of the nurse workforce over 45 years of age has increased from 29% in 1996 [5] to a plateau of around 51.7% of nurses in 2015 [6]. Yet, historically, few Australian registered nurses (<5%) remain in the workforce after age 60 [7].

Efforts to retain older nurses in the workforce need more knowledge about the issues that lead nurses to retire. Cross-occupational studies on the withdrawal from the workforce have tended to focus on intentions rather than actual behavior and have often been cross-sectional rather than longitudinal [8]. The continued emphasis in nursing research on turnover intentions is particularly concerning with one of the few large-scale, longitudinal studies on nurse turnover finding that intention was not associated with actual turnover, including actual retirement behavior, for nurses over the age of 45 in Italy [4]. Further, studies of nurse turnover have often included other forms of staff movement, such as retirement and dismissal, which could influence their results [9]. Retirement behavior needs to be specifically examined [10]. That is, studies are needed on the drivers of nurse retirement behavior specifically [11].

With a sizable subset of the population having years of relatively healthy life expectancy sometimes characterized as an encore period [12], older individuals can have ongoing participation in meaningful activities such as an extended paid working life. Consequently, this study will investigate predictors of actual retirement behavior, whether partial or complete, for nurses 45 years of age or older over a seven-year time span. The findings could inform the actions of nursing and health service managers wanting to avoid shortages by trying to keep nursing staff for longer by either delaying their complete retirement or facilitating the bridging to retirement through partial retirement.

### 1.1. The Life Course Perspective

The life course perspective is capable of describing the longitudinal adjustment process of retirement [13], where an individual’s life choices are made from among the available options that structure the life course [14]. These life choices represent the human agency made in situations constrained by social influences [15]. Life course theory is often used to explain retirement decisions through a sociocultural perspective on age, linking age to time, process and contextual constraints over the life course [14,16].

The life course is the coalescing of age-graded choices, considering family pathways and career paths that reflect the conditions for transitions such as retirement [17]. For example, the option of bridge employment allows older workers to choose their level of workforce participation [18], representing a more gradual transition from work to full retirement [19].

Within the framework of the life course perspective, the key driver is the worker’s age, along with the specific contexts impacting retirement such as the employees’ work-associated perceptions and social identity, as well as their social experience in other life spheres, such as marital life and family structure [13]. This study will operationalize key characteristics of life course theory that have also been found to be drivers of nurse retirement or retirement intentions, grouped in terms of age, organizational factors or personal factors (e.g., per [20]).

The study investigates the impacts of age and the work characteristics of employment type, conducts a global assessment of work conditions as represented by job satisfaction and then considers characteristics that overlap between the work and personal spheres including education and role, and the impact of health conditions on work ability, as well as a variety of health statuses (general, physical functioning and mental). The remaining social influences and reflections of identity that are investigated are in terms of partnered status and whether the nurse has resident children. This range of issues is investigated within the context of Australia and is briefly reviewed below.

Age can be a driver of retirement choices when individuals feel that social pressures leave them little choice but to retire [20], especially when they feel social constraints about retiring beyond a normative age [21]. The specific nature of age’s impact on retirement behavior has also been complicated by the definitions of retirement in some studies incorporating age; for example, where a nurse’s retirement may be classified as “early” based on normative criteria such as age 65. Therefore, studies need to separate out and consider age itself, which should be more effective when age is not part of the retirement outcome variables, such as through consideration of choices of partial or complete retirement behavior.

Beyond the impact of age, work-related factors such as the nature of work contracts and characteristics of work can impact remaining at work [22]. A variety of work characteristics have been found to often predict retirement decisions, where these characteristics can be assessed globally in terms of job satisfaction [20,23]. Further, the impact of work characteristics on retirement decisions begins to move across to more individually oriented factors because nursing is physically and emotionally demanding, where working as a nurse may lead to a reduction in work ability, which may lead to retirement [4].

Health and work ability represent a mix of issues that play a role in older nurses’ decisions about whether to remain in the workforce [20,23,24]. The ability to perform work decreases due to the individual’s general health status [11,22] and functional capacity as they age [25]. Further, health conditions are associated with increasing rates of workplace injury [26] and the high rate of injuries in nursing may limit work ability [27]. In general, reductions in health and/or work ability will tend to influence individuals toward retirement [22].

A key contextual issue that may determine whether these health and work ability issues impact retirement decisions is the national context, especially in terms of health and welfare support. The welfare and health support systems are somewhat similar to those in Sweden, where those contexts would have more health and welfare support settings in common, particularly relative to the US. At the moment, the Australian pension system does not have quite as much flexibility as the Swedish system (as described in [23]), but there is a means-tested pension system and relatively universal health care in Australia.

Another contextual issue to note is that in Australia the education level of a nurse would have a lot of commonality with their broad nursing category. That is, in some OECD countries such as France, Norway, Poland and Spain, the lower-level category of “associate professional nurse” does not exist, and all nurses are reported in the higher-level, “professional nurse” category. In countries such as Australia, Germany, the UK and the United States (US), the vast majority of nurses are considered to be higher-level nurses, and a minority are considered to be at the lower level [28]. In Australia, the higher-level registered nurses have at least a baccalaureate degree, whereas the associate professional nursing staff would usually have sub-baccalaureate qualifications.

The final set of issues considered as possible predictors of retirement behavior in this study are individual factors such as marital status [29] and family responsibilities [22]. The individual’s experience in their personal life sphere is important because, from a life course point of view, it may provide alternative salient identities to be carried into and possibly expanded upon in retirement [13].

Many of the variables above may impact the retirement decision over time. Yet, most prior retirement research has been cross-sectional (e.g., as summarized in [20], where most of the small number of longitudinal studies were over a time period of one year or less). From the life course perspective, retirement is a process that takes place over time and, therefore, should be studied longitudinally [15]. In particular, the effects of health on retirement decisions and behavior occur over time, with the health condition and/or work ability issues impacting workers over many years and work may worsen any health conditions, eventually making it difficult to continue to work.

### 1.2. The Present Study

This study investigates predictors of actual retirement behavior, whether partial or complete, for working nurses 45 years of age or older over a seven-year time span. The findings could inform the actions of nursing and health service managers wanting to avoid staffing shortages by trying to keep nurses at work by either delaying their complete retirement or facilitating the bridging to retirement through partial retirement. The study used a longitudinal quantitative design based on a survey of a nationally representative sample of the Australian population.

## 2. Materials and Methods

### 2.1. Study Participants

People participating in the Household, Income, and Labor Dynamics in Australia (HILDA) survey [30], a broad nationally representative survey, provided the data analyzed in this study. The University of Melbourne ethics approval number for the HILDA Survey Project is 1647030. HILDA uses a set of rules, in combination with an initial sample intended to be representative of all Australian households, to ensure that the panel retains its representativeness over time (see [30] for details).

The sample analyzed comprised those who had participated in the HILDA Survey in both 2012 (Time 1) and 2019 (Time 2), where the data were from the 2019 version released in 2021. Note that the detailed retirement behavior questions are only asked every four years and that the time period chosen was selected as to avoid any impacts of the global financial crisis, which had mostly settled in Australia by 2012, and before the potential impacts of COVID-19. Thus, the sample period enables a long-term (7-year), longitudinal, prospective analysis of retirement behaviors. Detailed information about the sampling processes and survey is available from the HILDA manual [30].

The sample comprised those respondents indicating that the main field of study for their highest completed qualification at Time 1 was in the field of nursing and were 45 years of age or older at Time 1. The next filter focused the sample on those respondents who at Time 1 were employed in relevant occupations as coded to the 2-digit Australian and New Zealand Standard Classification of Occupations. The vast majority of respondents indicated they were in the occupations of health professionals, health and welfare support workers, carers and aides, or social and welfare professionals. A small number of nursing-qualified participants were in the occupations of chief executives, general managers and legislators, specialist managers, business, human resource and marketing professionals, education professionals, legal, sports and personal service workers or office managers and program administrators. Checks were conducted on how long the nurses had been working in health occupations. After excluding some cases with nonsensical results (e.g., a few respondents indicated that they had changed occupation recently, but in checking their “previous” occupation, in all of the cases where data were available, the previous occupation was still nursing related), the remaining respondents had worked in their occupation for an average of 20.2 years at Time 1, confirming that the sample were long-term nurses and had a broadly similar profile to nurses of this age range for nurses across Australia [6].

Further, due to the small number of males (*n* = 20), only females were kept in the sample to be analyzed. The final sample had 190 female, nursing-qualified respondents matched over 7 years from Time 1 to Time 2, including 12 cases with missing values on one or more variables (check analyses on the cases with missing values are reported in the Results section).

### 2.2. Measures

Following the literature above, those variables indicating drivers that could have a prospective impact were measured at Time 1 and those variables that change slowly over time but may have been about to be initiated at Time 1 (e.g., marital status and education) were analyzed using Time 2 measures. For example, where someone may be one year of the way into a three year degree at Time 1, their job options at Time 2 would be a function of their education status at Time 2. Some of the variables appeared to have non-linear relationships with the target variables and were, consequently, coded to reflect those non-linear relationships.

*Age*: The nurses were asked to indicate their “Age last birthday at the 30 June 2012” for Time 1, and as at 30 June 2019 for Time 2. The Time 1 age was used to select the older nurses for the sample. The Time 2 age variable was used in the analyses.

*Employment Type—Full-time or Part-time Hours*: Participants indicated a number of hours in response to the question, “Including any paid or unpaid overtime, how many hours per week do you work on average over a usual 4-week period in all your jobs?” The hour ranges were grouped into being nominally full-time equivalent (35 h or more) or nominally part-time (less than 35 h).

*Job Satisfaction at Time 1*: Respondents were asked to provide a rating from 0—totally dissatisfied to 10—totally satisfied to indicate how satisfied or dissatisfied they were with their (main) job. The question was: “All things considered, how satisfied are you with your job?” The job satisfaction scores were very non-linear and were, consequently, coded into two groups. The distinctiveness of those scoring very highly satisfied meant that the variable could not be used as a linear variable. That is, for the analyses the responses were grouped into two categories, those scoring 0–8 (less satisfied) and those scoring 9–10 (quite satisfied).

*Work Ability at Time 1*: Participants were asked to indicate whether they had a long-term health condition, disability or impairment from a list they were shown that has lasted, or is likely to last, 6 months or more, restricts everyday activity and cannot be corrected by medication or medical aids. The list of conditions included: hearing problems, speech problems, a nervous or emotional condition that requires treatment, any condition that restricts physical activity or physical work, any mental illness that requires help or supervision, a long-term condition or ailment which is still restrictive even though it is being treated or medication is being taken for it, and any other long-term condition such as arthritis, asthma, heart disease, Alzheimer’s disease, or dementia. For the entire list overall, the response options were a single yes or no. Those respondents answering yes were also asked whether the condition limits the type or the amount of work they could do, with available responses of yes, no, or unable to do any work. Those answering yes to this follow-up question were asked to indicate how much their condition(s) limit(s) the amount of work they can do on a scale from zero, “not at all”, to 10, “unable to do any work”. Those respondents answering no to the initial question, or no to the limiting question, or 0 or 1 to the degree of impact question were grouped as 0—no work ability impact, whereas those going through the other filters that ended up scoring 2–10 on the degree of impact item were coded as 1—work ability impacted.

*Physical Functioning, General Health and Mental Health at Time 2*: The respondents’ scores for these three facets of health were measured using the SF-36 and the scale scores calculated following the scoring system outlined in [31], where each scale is transformed into a 0–100 index. All three health scales had a non-normal distribution and were transformed using the reverse-and-squared formula per [32], where the new health variables were equal to the square root of 101 minus the index score. Essentially, the scores were reversed and condensed a little in order to ensure the health variables met the assumptions of the later analyses.

*Education—Highest level of education achieved as of Time 2*: Respondents indicated the highest year of schooling they had completed and what qualifications had they completed, excluding hobby or recreation courses. Across the responses, the highest level of education was grouped such that the respondents with fewer years of education were in one group: year 11 of school or less; year 12; certificate 3 or 4; diploma or advanced diploma (coded as: less than or equal to/LTE diploma). The other group were those with a baccalaureate/bachelor’s degree, graduate diploma or postgraduate degree such as a master’s degree or doctorate (coded as: “bacc. and PG”).

*Partnered Status at Time 2*: The partnered or marital status of the respondent at Time 2 was obtained by asking: “Looking at [the options below], which of these best describes your current marital status?” The response options were: 1—married (in a registered marriage), 2—separated, but not divorced, 3—divorced, 4—widowed, 5—never married but living with someone in a relationship, and 6—never married and not living with someone in a relationship. The responses were grouped such that 1 and 5 were classed as partnered and the other options were grouped as not partnered/single. The codes combined within the single category all had similar relationships with the other variables and the retirement categories except for those who were widowed. However, there were so few widowed across the categories that including them in the partnered group did not change the significant results reported below, and consequently, the widowed were kept in the group to which they literally fitted—being single.

*Resident Children at Time 2*: The respondents were asked three questions about permutations of resident children potentially being present. The first question asked how many of the children they had ever had or adopted live in the household at least 50% of the time. The second question asked how many children live in a non-private dwelling (school, university hall of residence, institution) but spend the remainder of their time with you. The third question asked about the number of resident step/foster/grandchildren with no resident natural/adopted parent. If resident children were indicated for any of these three questions, the variable was coded yes; otherwise, as no.

*Retirement Status at Time 2*: Participants were asked: do you consider yourself to be completely retired from the paid workforce, partly retired or not retired at all? The answers were: completely retired, partly retired, not retired at all and not relevant—have never been in paid work. No respondents remaining in the sample indicated “Not Relevant”. The respondents were coded as being completely retired, partly retired, or not retired at all at Time 2.

## 3. Results

The final sample of 178 respondents, after excluding the 12 cases with any missing values, indicated at Time 2 that they were not retired at all (*n* = 117), partly retired (*n* = 20), or completely retired (*n* = 41). The pattern of responses of the variables across the retirement categories is detailed in Table 1. Analyses across all of the variables indicated that the missing values were missing completely at random (MCAR) with Little’s *p* = 0.633.

A multinomial regression analysis was performed using SPSS version 28 (Chicago, IL, USA) on the retirement status at Time 2 with age, part-time status, job satisfaction, physical functioning, general health, mental health, work ability, education, partnered status, and resident children as predictors. Following the best practice approach for multinomial regressions [33], the continuous variable of age was checked for curvilinearity using Box–Tidwell transforms and no curvilinear relationship was indicated. Therefore, age-squared, or other power variations of age, were not used in the model, and if included, did not add any information to the analyses, nor change the significant results presented in Table 2.

The multinomial regression had −2LL = 216.314, a significant improvement over the intercept-only base model of 306.024 (χ^2^(20) = 89.710, *p* < 0.001), indicating that the predictors, as a set, distinguished between the three retirement categories at Time 2. The Nagelkerke R-squared = 0.482, the Cox and Snell pseudo-R-squared = 0.396 and the McFadden R-squared = 0.293. The variables that were significant overall in the multinomial regression were age, physical functioning and employment type. The logit parameter estimates and the odds ratios are detailed in Table 2, where the comparison retirement category is being not retired at Time 2.

The above analyses were repeated with the inclusion of a pension age eligibility variable (being 65 and older), but that variable was not significant and none of the key relationships in Table 2 changed. There were four cases excluded from the above analyses who were unemployed at Time 1. When those unemployed cases were included in to the codes that they were most similar to (full-time hours and unsatisfied), the findings were the same as in Table 2.

To check any potential impact of missing values, a multinomial regression was conducted across 40 imputed datasets created using full information maximum likelihood estimation. Note that there were only 12 cases with missing values and all of the missing values were on the three health scales. The variables that were significant, or *p* < 0.10, in Table 2 remained significant or *p* < 0.10. The only slight change was that the partnered variable for completely retired becomes *p* < 0.10, where those with a partner had a tendency to be more likely to completely retire. Overall, the missing cases did not impact the pattern of significant results.

Age significantly separated both partly and completely retiring from not retired at all, where older nurses were more likely to choose one of the forms of retirement behavior. The only other variable that separated the completely retired from those not retired was that those nurses with part-time hours of work at Time 1 were more likely to completely retire.

The further variables that separated partial retirement from not being retired were job satisfaction, work ability and physical functioning. The direction of the relationships was such that those nurses with high job satisfaction were more likely to partly retire, those nurses with some impact on their work ability were more likely to partly retire and those nurses with better physical functioning were more likely to partly retire. There was a tendency (*p* < 0.10) for those with higher general health to partly retire.

## 4. Discussion

Age was the only variable that significantly predicted both forms of retirement behavior as distinct from not retiring. The consistency and strength of the age effects highlights the strength of social normative age effects (per [21]), where age reflects a set of age-graded influences [12] and can reflect a key constraint on retirement choices [20].

The remaining significant drivers differed depending on whether the outcome behavior was partial retirement or complete retirement. Working part-time hours at Time 1 was a predictor of being more likely to completely retire, suggesting that some nurses may have started bridging to retirement quite early, reflecting the historically low rates of nurses remaining in the workforce suggested previously in Australia (e.g., by [7]).

Managers and organizations may begin retaining more nurses through the option of partial retirement by addressing work ability and physical functioning issues, confirming the findings of [22] with quantitative, longitudinal data. At a minimum, such considerations could include (per [21]) health-promoting working environments, better inclusion of older workers in training at the workplace and adapting working conditions to workers, particularly to avoid injury [26,27]. Timely rehabilitation should be readily accessible to individuals with decreased work ability as part of actively managing health and work ability [22]. Similarly, this study’s findings support an increased emphasis on ergonomics in nursing, adjusting some tasks to age-related physical limitations and interests, and enabling lateral job movement among other examples of human resource management practices (per [23]).

An interesting complexity to these results is that both higher physical functioning and having impacted work ability were predictive of partial retirement. Reconciling these findings suggests a key set of issues that nurse and health service managers will have to consider as nurses age. That is, by considering the example of nurses who may have had both high physical functioning and impacted work ability, a possible resolution is that these nurses may have chronic diseases that the individuals are able to manage (e.g., with medication). Such a subset of workers are likely to be a growing proportion of the possible workforce and this complexity of health issues and work ability will need to be explicitly managed.

The inconsistent results of previous studies regarding the predictive power of job satisfaction has been clarified here by the results above indicating that high job satisfaction may keep nurses in the workforce as they partly retire, whereas previous studies may have indirectly combined partial and complete retirement. The contribution of job satisfaction is unlikely to be due to unintended overlaps with aggregate health measures, because the health and work ability variables were separately considered. Perhaps the most surprising implication of the significant job satisfaction result is that an attitude, admittedly a global assessment of one’s work, was able to predict retirement behavior seven years later.

The social identity variables of having children and marital status did not consistently distinguish between the work-retirement outcomes, although partnered status did indicatively suggest complete retirement. Similarly, education may occasionally influence nurses’ intentions to retire, but when it comes to actual retiring, other concerns are more prominent.

That is, proximal identity issues did not drive the retirement behaviors for nurses. The drivers against the age-oriented norms pulling nurses toward retirement were more about the nurses’ functionality and their attraction to, and opportunities at, work.

### Limitations

Another possible influence on the above results is that the non-significant variables may reflect selection effects, such as the “healthy worker effect” [34] where less healthy nurses leave the workforce early, in this case disproportionally before age 45, leaving healthier nurses behind in the workforce. However, the work ability variable was still able to be found to be a significant distinguishing characteristic of those nurses who completely retired, indicating there was ample opportunity for relationships between work ability, health-related variables and retirement behavior to be found. Other common limitations (e.g., common method variance) are less likely to apply due to the longitudinal nature of the study and the substantial time period between measurements.

Perhaps a key limitation, but also a useful consideration in understanding the pattern of results across studies, is the importance of the welfare context of the study. The Australian context has relatively universal health care as well as relatively broad safety net pension systems, supported by a superannuation system. The results of this study may be partly impacted by that welfare context and other studies may want to investigate retirement behaviors in other contexts, as noted below.

## 5. Conclusions

This study found that certain life course variables were predictors of partial retirement behavior and complete retirement behavior, respectively. A particular contribution was the successful delineation and analysis of partial retirement as a form of retirement behavior distinct from complete retirement. The delineation between the forms of retirement behavior was particularly clear in that most of the predictors of partial retirement were different to the predictors of complete retirement.

Working part-time hours at Time 1 being a predictor of completely retiring up to seven years later highlights the presence of bridging to retirement among these nurses and the need to consider the interconnectedness of partial and complete retirement. That is, those nurses working part-time may have effectively been partly retiring and bridging to complete retirement. Conversely, the nurses who were full-time at Time 1 are remaining at work, with similar likelihoods of remaining either at work full-time or part-retiring. Therefore, the challenge is, once the nurses move to partial retirement, to keep them in part-retirement by keeping them satisfied and having policies and practices that support those with work ability conditions that are manageable.

Considering finer gradations of retirement behavior, such as partial retirement, may open up a range of research topics and interventions that could help to retain nurses in the workforce. Further, this study focused on forms of actual retirement behavior rather than intentions. Another key contribution is the precedent for the prospective power of these drivers of retirement, where the variables predicted retirement behavior up to seven years later.

The consistency and nature of the relationship of age with both forms of retirement behavior reflects the normative expectations of society in general, but not the specific transitions to retirement such as those embodied in eligibility for the age pension. The social normative pressures associated with age could be considered to represent the tide, where nurses are pulled toward retirement. Fighting that tide could involve more widespread advocacy for policies that support partial retirement (although those policies may be more successful in the context of a pension system amenable to such partial retirement, such as in Sweden, as described in [23]).

At a more detailed level, fighting against the tide appears to be substantially about managing nurses’ functioning and capability. Nurse and health service managers could support nurses to stand against the social normative pressures toward retirement (until such social norms change) by managing the nurses’ physical functioning and work ability through mechanisms such as lateral transfers and ergonomics interventions (e.g., as advocated by [23]). Enabling nurses to bridge to complete retirement will retain older, experienced nurses in the workforce, at least for a while longer, allowing more time for supply side issues to be addressed. With an already notable and growing proportion of nurses facing the complexity of the impacts of chronic diseases and/or work on their health and work ability, managers will need to place more emphasis on explicitly managing these characteristics of their workforce.

## Figures and Tables

**Table 1:** The variables in the analyses by retirement category at Time 2.

		Retirement Status at Time 2		
Categorical Variables		Not Retired (*n*)	Partly Retired (*n*)	Completely Retired (*n*)
Employment type (Time 1)	- full-time hours	55	9	8
	- part-time hours	62	11	33
Job satisfaction (Time 1)	- less satisfied (0–8)	76	9	23
	- quite satisfied (9–10)	41	11	18
Work ability (Time 1)	- no impact	104	15	33
	- some impact	13	5	8
Education (Time 2)	- LTE ^1^ adv. dip./diploma	55	6	22
	- bacc. and PG	62	14	19
Partnered status (Time 2)	- not partnered	47	9	15
	- partnered	70	11	26
Has resident children (Time 1)	- yes	63	7	13
	- no	54	13	28
**Continuous Variables**		**Mean (Standard Deviation)**		
Age (Time 2)		58.79 (4.852)	64.45 (6.802)	66.27 (5.343)
Physical functioning index score (Time 1)		85.23 (16.331)	84.25 (19.687)	76.00 (19.377)
General health index score (Time 1)		71.18 (20.306)	69.10 (18.293)	66.02 (20.169)
Mental health index score (Time 1)		75.90 (17.192)	78.60 (11.843)	75.95 (15.261)

^1^ LTE = less than or equal to.

**Table 2:** Odds ratios of characteristics distinguishing between work-retirement end points.

Retirement Category (Ref: Not Retired at All at Time 2)	Partly Retired at Time 2			Completely Retired at Time 2		
Variables (Reference Category)	B (SE)	Odds Ratio	95% CI	B (SE)	Odds Ratio	95% CI
Age at Time 2	0.216 (0.057)	1.242 **	1.110–1.389	0.272 (0.051)	1.312 **	1.186–1.452
Employment type (Time 1), part-time hours (Ref: full-time hours)	−0.122 (0.584)	0.885	0.282–2.778	1.394 (0.532)	4.031 **	1.421–11.434
Job satisfaction (Time 1), quite satisfied (9–10) (Ref: less satisfied (0–8))	1.251 (0.622)	3.495 *	1.032–11.837	0.470 (0.500)	1.600	0.601–4.264
Physical functioning (Time 1), reversed with square root	−0.473 (0.205)	0.623 *	0.417–0.932	0.159 (0.174)	1.173	0.833–1.650
General health (Time 1), reversed with square root	0.400 (0.227)	1.491 ^†^	0.955–2.327	−0.091 (0.196)	0.913	0.622–1.340
Mental health (Time 1), reversed with square root	0.091 (0.184)	1.095	0.763–1.570	0.255 (0.170)	1.290	0.924–1.801
Work ability (Time 1), some impact (Ref: no impact)	1.741 (0.834)	5.702 *	1.113–29.215	0.474 (0.701)	1.607	0.407–6.344
Education (Time 2), bacc. and PG (Ref: LTE adv. dip)	0.804 (0.593)	2.234	0.699–7.139	−0.534 (0.485)	0.586	0.226–4.264
Partnered status (Time 2), partnered (Ref: not partnered)	0272 (0.577)	1.313	0.4245–4.064	0.944 (0.510)	2.569	0.946–6.978
Has resident children (Time 1), no (Ref: yes)	0.317 (0.602)	1.373	0.422–4.467	0.512 (0.503)	1.669	0.623–4.473
Intercept	−17.580 (3.917) **			−21.176 (3.606) **		

Note: † <0.10, * <0.05, ** <0.01. SE = standard error, CI = confidence interval, GTE = greater than or equal to, LTE = less than or equal to, Ref. = reference/comparison category.

## Data Availability

The HILDA data are available and managed by the Melbourne Institute of Applied Economic and Social Research at The University of Melbourne (https://melbourneinstitute.unimelb.edu.au/hilda). The data used above were from the Wave 20 Release.
